# Microsoft Kinect-based Continuous Performance Test: An Objective Attention Deficit Hyperactivity Disorder Assessment

**DOI:** 10.2196/jmir.6985

**Published:** 2017-03-20

**Authors:** David Delgado-Gomez, Inmaculada Peñuelas-Calvo, Antonio Eduardo Masó-Besga, Silvia Vallejo-Oñate, Itziar Baltasar Tello, Elsa Arrua Duarte, María Constanza Vera Varela, Juan Carballo, Enrique Baca-García

**Affiliations:** ^1^ Universidad Carlos III Department of Statistics Madrid Spain; ^2^ Hospital Universitario Fundación Jiménez Díaz Child and Adolescents Service Madrid Spain; ^3^ Goal Systems Co Madrid Spain; ^4^ Hospital Universitario Gregorio Marañón Child and Adolescents Service Madrid Spain; ^5^ Columbia University New York, NY United States; ^6^ IIS-Jimenez Diaz Foundation Department of Psychiatry Madrid Spain; ^7^ Universidad Autónoma Madrid Spain; ^8^ Hospital Universitario Rey Juan Carlos Móstoles, Madrid Spain; ^9^ Hospital Universitario Infanta Elena Valdemoro, Madrid Spain; ^10^ Hospital General de Villalba Villalba, Madrid Spain; ^11^ CIBERSAM Madrid Spain

**Keywords:** kinect, attention deficit hyperactivity disorder, continuous performance test, impulsivity, hyperactivity

## Abstract

**Background:**

One of the major challenges in mental medical care is finding out new instruments for an accurate and objective evaluation of the attention deficit hyperactivity disorder (ADHD). Early ADHD identification, severity assessment, and prompt treatment are essential to avoid the negative effects associated with this mental condition.

**Objective:**

The aim of our study was to develop a novel ADHD assessment instrument based on Microsoft Kinect, which identifies ADHD cardinal symptoms in order to provide a more accurate evaluation.

**Methods:**

A group of 30 children, aged 8-12 years (10.3 [SD 1.4]; male 70% [21/30]), who were referred to the Child and Adolescent Psychiatry Unit of the Department of Psychiatry at Fundación Jiménez Díaz Hospital (Madrid, Spain), were included in this study. Children were required to meet the Diagnostic and Statistical Manual of Mental Disorders (DSM-5) criteria of ADHD diagnosis. One of the parents or guardians of the children filled the Spanish version of the Strengths and Weaknesses of ADHD Symptoms and Normal Behavior (SWAN) rating scale used in clinical practice. Each child conducted a Kinect-based continuous performance test (CPT) in which the reaction time (RT), the commission errors, and the time required to complete the reaction (CT) were calculated. The correlations of the 3 predictors, obtained using Kinect methodology, with respect to the scores of the SWAN scale were calculated.

**Results:**

The RT achieved a correlation of -.11, -.29, and -.37 with respect to the inattention, hyperactivity, and impulsivity factors of the SWAN scale. The correlations of the commission error with respect to these 3 factors were -.03, .01, and .24, respectively.

**Conclusions:**

Our findings show a relation between the Microsoft Kinect-based version of the CPT and ADHD symptomatology assessed through parental report. Results point out the importance of future research on the development of objective measures for the diagnosis of ADHD among children and adolescents.

## Introduction

Attention deficit hyperactivity disorder (ADHD) is the most common childhood neurodevelopmental disorder with an estimated prevalence of around 6% [1]. ADHD is characterized by a pattern of hyperactivity-impulsivity, and inattention. According to the fifth edition of the Diagnostic and Statistical Manual of Mental Disorders (DSM-5), these symptoms usually appear before the age of 12 years and, in order to be considered ADHD, must be present in 2 or more settings such as the school or at home [2]. They can be associated with worse academic performance [3] and cognitive difficulties in the future [4]. Moreover, ADHD increases the risk of developing other psychiatric or psychological disorders [5,6].

Early ADHD diagnosis is of paramount importance in order to minimize the negative cognitive, emotional, academic, and social effects associated with this condition and to restore the child’s functioning. However, obtaining an accurate ADHD diagnosis is complicated, as most of the psychiatric and psychological criteria are based on clinical evidence. Questionnaires and scales are probably the most common assessment instruments. The Strengths and Weaknesses of ADHD symptoms and Normal behavior (SWAN) rating scale [7], the diagnosis criteria of the DSM-5 [2] or the ADHD rating scale [8] are some of the different tools for evaluating this medical condition. However, both questionnaires and scales present several inconveniences. For instance, the veracity and accuracy of the answers is not granted [9]. Frazier et al [10] showed that individuals might be tempted to mimic ADHD symptoms to justify a failure at school or work. Receiving a disability status to obtain certain benefits or getting access to stimulant drugs are other two common causes for people to conscientiously exaggerate their symptoms [11]. Conversely, Middeldorp et al [12] have exposed the parental psychological difficulties for internalizing ADHD symptoms, which may influence the answers of the questionnaires. In addition, questionnaires and scales are unsuitable for repeated use due to learning issues [13]. Finally, it has been pointed out that scales suffer from cultural biases [14].

In order to alleviate these weaknesses, new instruments have been developed for assessing ADHD. Currently, the continuous performance test (CPT) is one of the most popular tools for evaluating ADHD [15]. In this test, the examinee is required to press the spacebar in a keyboard as fast as possible every time certain characters appear on a computer screen. If a forbidden character, referred to as an X-stimulus appears on the screen, the examinee must inhibit the reaction. Several studies have demonstrated that CPT has the ability to differentiate children with and without ADHD [16]. CPT measures attention problems with an overall index from reaction time (RT), RT variability, and omissions [17]. In addition, impulsivity is measured by RT and commissions [17]. Notwithstanding its success, some researchers [18] have questioned its utility as a mechanism for differential diagnosis of ADHD. In particular, they found that whereas children with reading disorders have high CPT scores, those with ADHD did not show significantly different scores from clinical controls.

In order to increase the accuracy of the CPT, it has been proposed to additionally collect predictors of the participant’s bodily movements during the execution of the test. One common approach consists of the use of accelerometer-based devices (actigraphy and inertial measurement units, IMUs) [19,20]. However, accelerometer-based devices appear to require a prolonged interval of acquired data in order to make an accurate prediction; moreover, they only record movement in 1 or 2 locations on the body. In addition, the intrusiveness of the method is a disadvantage. Studies combining actigraphy with CPT suggest a better accuracy of ADHD diagnosis compared with the use of a standard CPT alone [21].

A second group of techniques is based on the use of infrared motion trackers (McLean Motion Analysis Test and QbTest), small reflectors attached to the child’s body for the duration of the test to aid ADHD assessment [22,23]. Studies based on these tracking systems have reported that combined CPT and infrared motion analysis differentiates ADHD children from normal controls [24]. However, these systems are time-locked and although they are able to record the path of movement, they are not yet able to integrate the movement data that are collected by different sensors [25]. As the previously described method, this technique also presents an intrusiveness problem.

One of the devices that are attracting more attention is the Microsoft Kinect. It consists of a standard RGB camera, an infrared sensor, an infrared projector, and a set of 4 microphones. Since its appearance in 2010, several studies have attempted its use for cognitive assessment. For instance, Qiu and Helbig found a statistically significant relation between the corporal posture captured with the Kinect camera and the mental load required to complete 4 different tasks [26]. They observed that the participants’ head was close to the screen and the body trunk was tilted forward when the task was more cognitively demanding. In a different work, Yu et al suggested the possibility of detecting children’s abnormal behavior using Kinect [27]. Likewise, Stanley proposed to estimate attention levels combining body posture and head orientation [28], aiming at improving the ADHD diagnosis procedure. In a recent work, Li et al [25] used the Kinect for monitoring the intensity of children’s movement during a Go or No-Go task in order to attain a better characterization of ADHD. They proposed an initial measure of total movement intensity, indicated by the number of displaced pixels in the silhouette of the individual in 2 consecutive frames. They apply a Fourier transform to the original time-domain signal to produce a Movement Intensity Distribution which itself is decomposed in 15 alternative nonoverlapping 1 Hz frequency bands. Their results show that each of these bands is a predictor capable of discriminating ADHD children from healthy controls. Unlike the previous studies which were limited to discriminating between two groups (ADHD and controls), these authors found that some of the frequency bands were correlated with the score obtained in the Clinical Global Impression Scale and the ADHD-Rating Scale IV. This result is interesting because none of the traditional measures obtained with the Go or No-Go task, for example, RT or commissions, was correlated with the scores of these psychometrical scales.

In this paper, we proposed a new method for evaluating the severity of ADHD. The method builds in a previous study by Delgado-Gomez et al [29], where the CPT and the Kinect device are combined in order to assess the impulsivity of 22 university students. Results of that study showed that by replacing the keyboard input with body movements, it was possible to obtain a more precise impulsivity assessment. In particular, using a Kinect device, it was found that if the examinee reacts to the stimuli by raising the dominant hand instead of pressing the spacebar, the impulsivity assessment is more accurate than with the sole use of the keyboard because the device is capable of detecting inhibited reactions. We hypothesized that this methodology will provide a more objective evaluation of ADHD to help clinicians in their diagnosis. In particular, the reaction time and number of commissions are good indicators of the severity of the ADHD.

## Methods

### Subjects

Thirty children, aged 8-12 years (10.3 [SD 1.4]; male 70% [21/30]), who were referred to the Child and Adolescent Psychiatry Unit of the Department of Psychiatry at Fundación Jiménez Díaz Hospital (Madrid, Spain), were included in this study. All participants met the DSM-5 criteria for ADHD [2]. Individuals with ADHD may present both inattention and hyperactivity or impulsivity or only one pattern may be predominant. These 3 traits of ADHD are commonly referred to combined-type, inattentive-type, and hyperactive or impulsive-type (50% [15/30] of the patients were diagnosed as inattentive subtype; 43% [13/30] as hyperactive or impulsive subtype; and 7% [2/30] as combined subtype).

### SWAN Scale

The SWAN scale [7] is the diagnosis instrument used in this study to compare the performance of the proposed method with respect to the current clinical diagnose. It is composed of 18 items based on the DSM-5 criteria for ADHD diagnosis which measure positive attention and impulse regulation behaviors in the normal population. The scale is made up of 3 factors. The first factor is associated to inattention and comprises the first 9 items. The following 6 items characterized the hyperactivity factor. The last factor, which comprises the last 3 items, measures impulsivity. In the standard form, each item is scored from −3 to +3 (below average to above average), where 0 is “normal” and based on the population average. This work uses a Web-based mental state tracking e-tool [30] where each item is scored from 0 to 100.

### Kinect CPT

In this study, we use a version of the Microsoft Kinect-based CPT proposed by Delgado-Gomez et al [29]. Originally, the Kinect-based CPT has duration of approximately 15 min. During this time, 360 letters appear sequentially on a screen with a time separation of 1, 2, or 4 s. Each time that a non–X-stimulus appears, the participant has to raise his dominant hand as soon as possible and return it to the rest position. When an X-stimulus appears, the examinee must inhibit any reaction.

The version used in this study includes some modifications to avoid the inconveniences reported by the authors. Namely, the duration of the original Kinect-based CPT resulted exhausting for the participants. Moreover, the gap of 1 s between some stimuli was too short for the examinee to be able to complete the action before the appearance of the next character. In order to tackle these inconveniences, the version used in this study takes only 3 min during which 60 letters are presented. Moreover, the time separation between stimuli is modified to 2, 3, or 5 s. A subset of 12 characters is X-stimuli and the remaining 48 are non–X-stimuli.

The RT for the non–X-stimuli, measured as the time elapse between the appearance of the stimulus and the moment in which the participant starts to raise the dominant hand, is calculated. A commissions’ index was calculated for the X-stimuli according to Delgado-Gomez et al. Concisely, it is computed as the ratio of the length of the hand displacement during the X-stimulus and the maximum displacement after the appearance of the previous and posterior stimuli. In addition to these 2 measures, the time required for completing the action of rising and returning the hand to the rest position is also calculated for the non–X-stimuli. Each participant is characterized with the median of the 48 RTs and 48 CTs and the median of the 12 commission indices. The proposed technique is illustrated in the Multimedia Appendix 1.

### Ethics Procedures

Parents or guardians were required to sign an informed consent after been explained the project in detail. Participants provided assent. The consent and assent forms and the study protocol were reviewed and approved by the Institutional Review Board of Fundación Jiménez Díaz Hospital. All procedures performed were conducted in accordance with the ethical standards of the institutional and national research committee and with the 1964 Helsinki declaration and its later amendments or comparable ethical standards.

During the experiment, a trained psychiatrist accompanied each of the 10 patients while they conducted the Kinect-based CPT. While each child was performing the Kinect-based test, the corresponding parent or guardian filled the SWAN scale.

## Results

**Figure 1:**
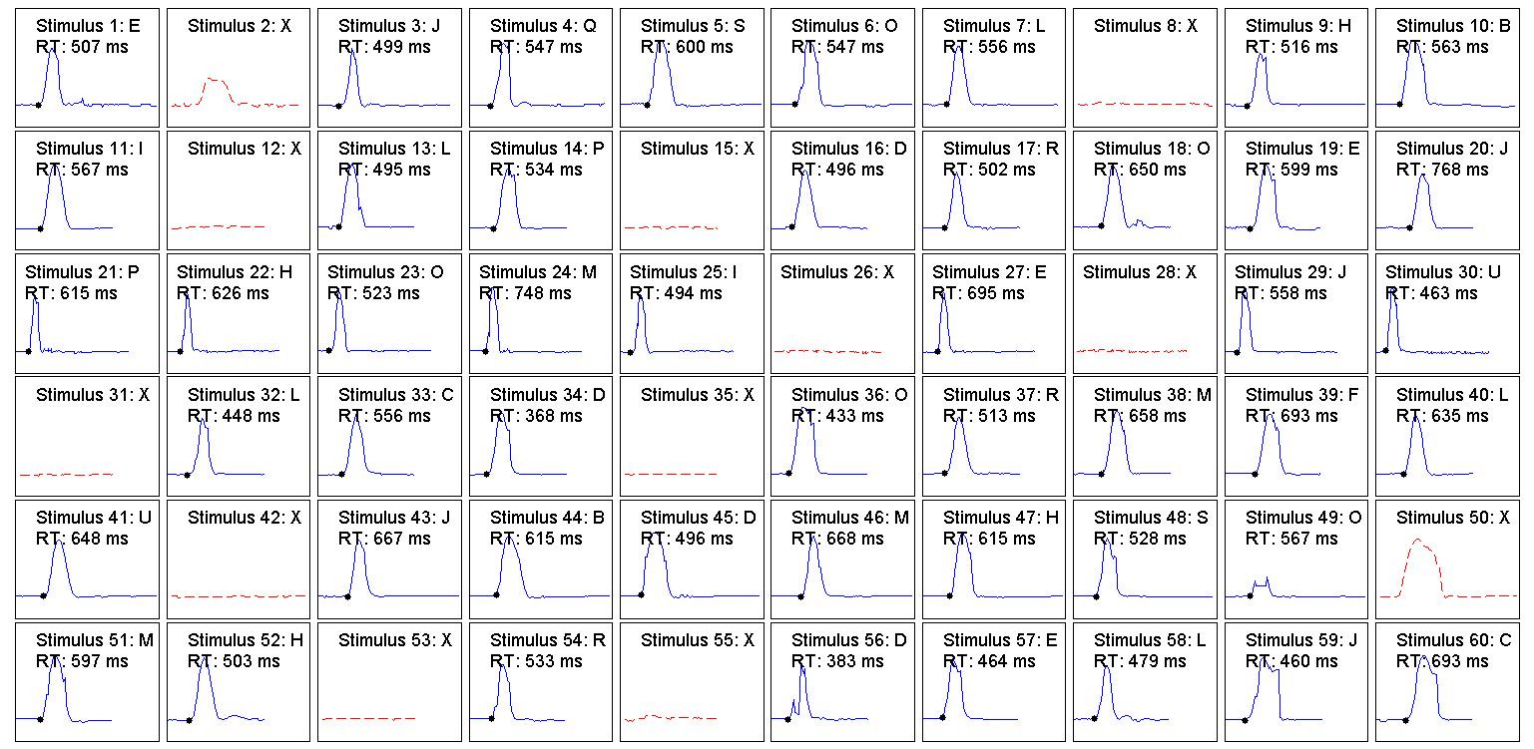
Performance of a participant with low scores in the impulsivity and inattention factors of the SWAN scale during the test. SWAN: Strengths and Weaknesses of ADHD Symptoms and Normal Behavior.

**Figure 2:**
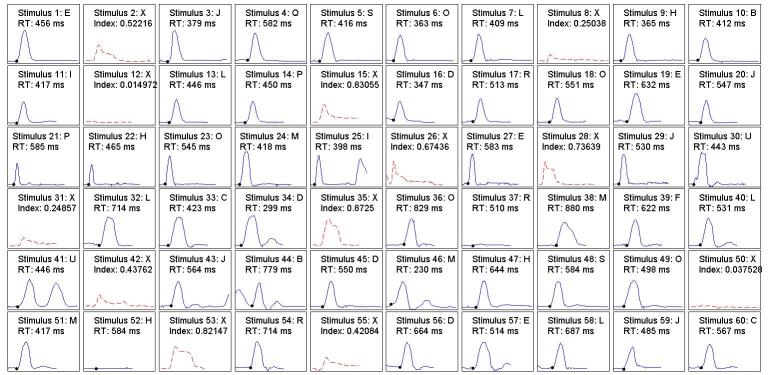
Performance of a participant with high scores in the impulsivity and inattention factors of the SWAN scale during the test. SWAN: Strengths and Weaknesses of ADHD Symptoms and Normal Behavior.

Before presenting the main results of the experiment, we present the complete performances of 2 examinees in Figures 1 and 2. Figure 1 shows the reaction to each of the 60 stimuli of a participant with a low score in the SWAN scale for impulsivity and inattention. It can be appreciated that there is only 1 commission, 1 inhibited commission (stimuli 50 and 2, respectively), and 1 partial omission (stimulus 49). It can also be seen that in most cases the RT was higher than 500 ms. Figure 2 shows the results of a child with high score in the SWAN impulsivity and inattention scale. We can see that, unlike the previous case, this patient incurred a much larger number of commissions (eg, stimuli 15, 35, and 53): 2 inhibited commissions (8 and 31) and 2 omissions (37 and 52). The occurrence of multiple reactions could also be noted (eg, stimuli 25, 41, and 44). There was 1 case where the reactor started before the stimulus had appeared (stimuli 43-44). Regarding the RT, in nearly 46% of cases, the reactions of this patient were less than 500 ms.

Table 1 shows the statistical summary of our findings. The first row shows the correlations of the RT median with respect to the inattention, hyperactivity, and impulsivity scores provided by the SWAN scale. It can be seen that impulsivity shows a statistically significant negative correlation, clearly indicating that individuals with high impulsivity tend to have low reaction times. A similar result can be observed for hyperactivity, suggesting that hyperactive patients also tend to show low reaction times. There was no evidence suggesting a strong correlation between RT and inattention.

Regarding the commission, the second row in Table 1 shows the correlation of the median of the commission indices with respect to each of the 3 factors of the SWAN scale. It can be observed that the values are positive for the impulsivity factor; and nearly 0 for hyperactivity and inattention. The results suggest that impulsive patients have more difficulties in inhibiting their reactions.

**Table 1:** Correlation of the median of the RT and commissions with respect to the 3 factors of the SWAN scale (inattention, hyperactivity, and impulsivity).

Explanatory variables	Inattention	Hyperactivity	Impulsivity
RT^a^	−.11 (*P*=.53)	−.29 (*P*=.11)	−.37 (*P*=.04)
Commission	−.03 (*P*=.83)	.01 (*P*=.92)	.24 (*P*=.20)

^a^RT: reaction time.

## Discussion

In this paper, we propose an innovative approach to assess the severity of ADHD. With this aim, we have developed a completely Kinect-based version of the CPT that allows tracking the complete movement of the examinees’ dominant hand, instead of only the pulse of the spacebar in the keyboard, during the performance of the test. With this tool, the examiner can not only record commissions, but also detect inhibited movements and obtain a more accurate measure of the reaction times. It is important to mention that the proposed technique differs from other available visually aided approaches, in that they still rely on the keyboard pulsation, whereas in our methodology the individual’s hand takes the place of the spacebar.

In particular, our technique computes the RT and the commission indices for each participant. In order to assess their accuracy, these 2 predictors are compared with each of the 3 factors (inattention, hyperactivity, and impulsivity) of the SWAN scale. The results show that the proposed approach is capable of assessing hyperactivity and impulsivity degrees of individuals diagnosed with ADHD. Regarding the commissions, notwithstanding that the correlation with impulsivity is larger than .2, this value is not statistically significant. A possible explanation for this, as suggested by the much larger values observed in a previous work conducted with healthy population, is that the increase in the inter-stimuli time allows the patient to take a more relaxed approach to the test as then mental load is reduced. Future work will consider reducing these times. These results are in concordance with the ones reported in the literature: in particular, (1) there is a positive correlation between impulsivity and the number of commissions [31]; and (2) it is not possible to establish a correlation between inattentiveness and the number of commissions [17].

Our results have also allowed us to establish a correlation between the RT and impulsivity or hyperactivity. This result is also consistent with the available literature. Moreover, this is an interesting result as Li et al [25] did not find a relation between the traditional CPT reaction time and the severity of ADHD as measured by the ADHD-RS IV scale.

Although our results have shown the proposed approach as a promising research line, its main limitation is the small sample size, consequence of the difficulty of enrolling participants with specific characteristics (children with the age of 8 to 12 years diagnosed with ADHD). Therefore, it is the intention of the authors to replicate the experiment with a larger number of individuals, where the different ADHD subgroups are well represented.

 

Despite the reduced sample size, our results point out our proposed methodology as an alternative for overcoming the limitations of the current ADHD assessment instruments based mainly on scales and questionnaires. From the clinical prospective, it can be an important aid in differentiating ADHD subtypes. It could also be useful for evaluating the impact of parent’s (potentially biased) perception of the child’s symptoms on the scales results [12]. Moreover, even though this work has focused on the movement of the dominant hand, the potential of the proposed technique is much greater. The analysis of other body parts, which are not directly involved in the reaction to stimulus such as body leaning, legs movement, or body configuration, may provide other discriminative predictors that could improve ADHD characterization and provide more objective diagnoses. In addition, although the current work has focused on the body, face analysis can also be incorporated in future studies.

## Appendix

Multimedia Appendix 1 Video demonstrating the working of the proposed technique.

## Abbreviations

ADHD: attention deficit hyperactivity disorder
CPT: continuous performance test
RCT: reaction time
SWAN: Strengths and Weaknesses of ADHD Symptoms and Normal Behavior
